# Transgenic rice seed expressing flavonoid biosynthetic genes accumulate glycosylated and/or acylated flavonoids in protein bodies

**DOI:** 10.1093/jxb/erv429

**Published:** 2015-10-04

**Authors:** Yuko Ogo, Tetsuya Mori, Ryo Nakabayashi, Kazuki Saito, Fumio Takaiwa

**Affiliations:** ^1^Transgenic Crop Research and Development Centre, National Institute of Agrobiological Sciences (NIAS), Tsukuba, Ibaraki, Japan; ^2^RIKEN Center for Sustainable Resource Science, 1-7-22 Suehiro-cho, Tsurumi-ku, Yokohama 230-0045, Japan; ^3^Graduate School of Pharmaceutical Sciences, Chiba University, 1-8-1 Chuo-ku, Chiba 260-8675, Japan

**Keywords:** Flavonoid, genetically modified plant, glycosylation, liquid chromatography-mass spectrometry, metabolome, rice, secondary metabolite.

## Abstract

Plant-specialized (or secondary) metabolites represent an important source of high-value chemicals. In order to generate a new production platform for these metabolites, an attempt was made to produce flavonoids in rice seeds. Metabolome analysis of these transgenic rice seeds using liquid chromatography-photodiode array-quadrupole time-of-flight mass spectrometry was performed. A total of 4392 peaks were detected in both transgenic and non-transgenic rice, 20–40% of which were only detected in transgenic rice. Among these, 82 flavonoids, including 37 flavonols, 11 isoflavones, and 34 flavones, were chemically assigned. Most of the flavonols and isoflavones were *O*-glycosylated, while many flavones were *O*-glycosylated and/or *C*-glycosylated. Several flavonoids were acylated with malonyl, feruloyl, acetyl, and coumaroyl groups. These glycosylated/acylated flavonoids are thought to have been biosynthesized by endogenous rice enzymes using newly synthesized flavonoids whose biosynthesis was catalysed by exogenous enzymes. The subcellular localization of the flavonoids differed depending on the class of aglycone and the glycosylation/acylation pattern. Therefore, flavonoids with the intended aglycones were efficiently produced in rice seeds via the exogenous enzymes introduced, while the flavonoids were variously glycosylated/acylated by endogenous enzymes. The results suggest that rice seeds are useful not only as a production platform for plant-specialized metabolites such as flavonoids but also as a tool for expanding the diversity of flavonoid structures, providing novel, physiologically active substances.

## Highlight

Glycosylated and/or acylated flavonoids in transgenic rice seeds were characterized by metabolome analysis, suggesting that ectopic expression of flavonoid biosynthetic enzymes can be used as a tool to expand their structural diversity.

## Introduction

Plant-specialized (or secondary) metabolites are an important source of high-value chemicals with pharmacological properties. Chemical synthesis is difficult due to their complex structures so these compounds generally have to be isolated from natural biological sources despite the difficulties in extraction and purification. There is, therefore, growing interest in producing plant-specialized metabolites in genetically modified organisms. Genetic engineering of plants for the production of specialized metabolites, which could serve as a renewable and sustainable production platform, would help fulfill the demands for these products.

Flavonoids are widespread, specialized plant metabolites that have diverse health-promoting benefits and are one of the target classes of metabolites that can be produced in genetically modified organisms. More than 8 000 compounds with a flavonoid structure have been identified in plants. The large number of different flavonoid structures arises from the various combinations of multiple hydroxyl groups, methyl groups, glycosides, and acylated group substituents on the basic benzo-γ-pyrone backbone (C6-C3-C6; Hodek *et al.*, 2002). There are two types of glycosylation, *O*- and *C*-linked glycosylation, which are susceptible and resistant to hydrolysis, respectively. Flavonoids represent a group of phytochemicals exhibiting a wide range of biological activities, such as anti-bacterial, anti-viral, anti-inflammatory, cardioprotective, neuroprotective, cancer preventative, anti-allergic, and estrogenic effects, arising mainly from their antioxidant properties and their ability to modulate several enzymes or cell receptors (Cornwell *et al.*, 2004; Dixon, 2004; García-Lafuente *et al.*, 2009; Oyama *et al.*, 2009; Perez-Vizcaino and Duarte, 2010; Shukla and Gupta, 2010; Gasiorowski *et al.*, 2011; Larson *et al.*, 2012). Flavonoids are divided into several major classes, such as flavanones, flavonols, isoflavones, and flavones, and the health-promoting effects differ among the individual classes. In addition, modifications to flavonoid aglycones, such as hydroxylation, methylation, glycosylation, and acylation, affect properties such as their stability, solubility, and biological activity depending on the type and position of the conjugation. These modifications can improve and increase the diversity of the drug-related properties of flavonoids (Takahama, 1986; Calias *et al.*, 1996; Fossen *et al.*, 1998; Makris and Rossiter, 2000; Ishihara and Nakajima, 2003; Buchner *et al.*, 2006; Pichersky *et al.*, 2006; Tian *et al.*, 2009; Chen *et al.*, 2011; Plaza *et al.*, 2014).

The basic biosynthetic pathways leading to the production of the core flavonoid skeletons have been well-characterized in terms of the molecular, genetic, and biochemical mechanisms of the enzymes involved (Dixon and Pasinetti, 2010). Several glycosyltransferases that catalyse the transfer of a sugar moiety to flavonoids and several enzymes that catalyse acyl transfer reactions have also been identified (St-Pierre and De Luca, 2000; Kim *et al.*, 2009; Falcone Ferreyra *et al.*, 2013; Li *et al.*, 2014). After synthesis, flavonoids are sequestered and compartmentalized into different subcellular locations, including vacuoles, vesicles, and nuclei or they are exported to the cell exterior (Mueller and Walbot, 2001; Saslowsky *et al.*, 2005; Taylor and Grotewold, 2005; Yazaki, 2005). Flavonoids located in different subcellular compartments are plant species-, cell type-, and flavonoid-specific. Glycosylation and acylation are important for the transport and storage of many flavonoids (Vaistij *et al.*, 2009; Bowles and Lim, 2010; Hassan and Mathesius, 2012; Francisco *et al.*, 2013). Several molecular players involved in the sequestration of flavonoids have been identified and associated with several models such as membrane transporter-mediated transport and vesicle-mediated transport (Kitamura, 2006; Grotewold and Davies, 2008; Zhao and Dixon, 2009). However, the mechanisms underlying flavonoid transport continue to be a matter of debate.

Rice seeds have recently received attention as a production platform for human pharmaceuticals such as therapeutic proteins and peptides. This system has many advantages in terms of cost-effectiveness, scalability, safety, product stability, and productivity (Wakasa and Takaiwa, 2013). Transgenic rice has previously been generated that accumulated high amounts of flavonols, isoflavones or flavones in seeds (Ogo *et al.*, 2013). This suggests that rice seed is a suitable bioreactor for producing proteins, as well as specialized metabolites such as flavonoids, because of the presence of organelles for flavonoid accumulation and the limited inhibition of endogenous enzymes after the production of flavonoids by exogenous enzymes. In these transgenic rice plants, genes involved in flavanone synthesis [*phenylalanine ammonia lyase* (*PAL*) and *chalcone synthase* (*CHS*)], as well as the genes involved in flavonol synthesis [*flavanone 3-hydroxylase* (*F3H*) and *flavonol synthase* (*FLS*)], *isoflavone synthase* (*IFS*), and *flavone synthase* (*FNS*), respectively, were expressed in a seed-specific manner. Although most flavonoids synthesized in transgenic rice are derived from the intended aglycone, i.e. kaempferol, genistein, and apigenin/chrysoeriol/tricin, respectively, these flavonoids appear to be differently glycosylated and acylated (Ogo *et al.*, 2013).

The development of liquid chromatography tandem mass spectrometry (LC-MS/MS) analysis has enabled the intensive characterization and quantification of flavonoids in various plant species. Recently, nearly 100 flavonoids were characterized and/or quantified in rice using an LC-MS-based metabolomics method (Dong *et al.*, 2014; Galland *et al.*, 2014; Kusano *et al.*, 2014; Yang *et al.*, 2014). In the present study, metabolome analysis was performed using liquid chromatography-photodiode array-quadrupole time-of-flight mass spectrometry (LC-PDA-QTOF-MS) to investigate flavonoid accumulation in transgenic rice seeds and many flavonoids could be chemically assigned by exploring flavonoid modifications such as glycosylation and acylation.

## Materials and methods

### Extraction of flavonoids

Flavonoids were extracted from whole seeds, endosperm, and embryos for LC-PDA-QTOF-MS analysis with three biological replicates as previously described by Ogo *et al.* (2013) with minor modifications. Flavonoids in whole seeds and endosperm were extracted from pulverized samples with 50 vols of water containing 0.1% formic acid per sample weight, while flavonoids in embryos were extracted from 20–30mg powder samples with 1ml of water containing 0.1% formic acid. This process involved vigorous vortexing, 30min of sonication, and incubation with shaking for 4h at room temperature. After the mixture was centrifuged, the supernatant was stored (as a water extract) and the residue was resuspended in equal volumes of methanol containing 0.1% formic acid. After 4h shaking at room temperature, the supernatant was stored (as a methanol extract). The water and methanol extracts were dried and mixed. The dried pellets from whole seeds and endosperm were resuspended in 7.5 times of 80% methanol containing 2.5 μM lidocaine and 10-camphour sulphonic acid to sample weight, while those from embryos were resuspended in 150 μl of 80% methanol. These samples were filtered through 0.2 μm filters before performing LC-PDA-QTOF-MS.

### LC-PDA-QTOF-MS analysis

The extracts (1 μl) were analysed using LC-PDA-QTOF-MS (LC, Waters Acquity UPLC system; MS, Waters Xevo G2 Q-Tof). The analytical conditions were as follows: LC column, Acquity bridged ethyl hybrid C18 (1.7 μm, 2.1×100mm, Waters); solvent system, solvent A (water including 0.1% formic acid) and solvent B (acetonitrile including 0.1% formic acid); gradient programme, 90% A/10% B at 0min, 90% A/10% B at 0.1min, 80% A/20% B at 25min, 0% A/100% B at 25.1min, 0% A/100% B at 27.5min, 90% A/10% B at 27.6min, and 90% A/10% B at 30.0min; flow rate, 0.3ml min^–1^; column temperature, 40 °C; photodiode array, 200–600nm; flavonoid detection, 340nm; MS detection: capillary voltage, +3.0 keV; cone voltage, 25.0V; source temperature, 120 °C; desolvation temperature, 450 °C; cone gas flow, 50 l h^–1^; desolvation gas flow, 800 l h^–1^; collision energy, 6V; mass range, *m/z* 50–1500; scan duration, 1.0 s; inter-scan delay, 0.014 s; data acquisition, centroid mode; polarity, positive; Lockspray (Leucine enkephalin): scan duration, 1.0 s; and inter-scan delay, 0.1 s. MS/MS data were acquired in ramp mode under the following analytical conditions: (i) MS: mass range, *m/z* 50–1500; scan duration, 0.1 s; inter-scan delay, 0.1 s; data acquisition, centroid mode; and (ii) MS/MS: mass range, *m/z* 50–1500; scan duration, 0.02 s; inter-scan delay, 0.014 s; data acquisition, centroid mode; and collision energy, ramped from 10V to 50V. In this mode, MS/MS spectra of the top 10 ions (>1 000 counts) in a MS scan were automatically obtained. If the ion intensity was less than 1 000, MS/MS data acquisition was not performed, and the next 10 ions were examined. Data acquisition and processing were performed using MassLyxs 4.1.

Peaks with intensity greater than 2 500 (noise level) were recorded. A peak with an intensity of less than 2 500 was transposed to an intensity of 2 500 so that it was not subject to the influence of noise. The intensity values of the peaks were divided by those of lidocaine ([M+H]^+^, *m/z* 235.1804) for normalization. The peaks from embryo samples were further divided by the magnification of each sample weight to 20mg for the normalization of sample weight. The normalized intensities of three biological replicates were averaged. The processed data were subjected to Principal Component Analysis (PCA) by SIMCA-P 11.5. Hierarchical clustering was performed by Centroid Linkage Clustering in Cluster 3.0 (http://bonsai.hgc.jp/~mdehoon/software/cluster/software.htm) using the peak-normalized value (i.e. the intensity of each peak was divided by the median intensity of all lines/tissues for each peak). Levels of metabolite identification were determined as defined by the Metabolomics Standards Initiative (Sumner *et al.*, 2007). For Venn diagrams, the peak data from line 19 of flavonol rice, line 12 of isoflavone rice, and line 20 of flavone rice were used.

### Expression profiling of OsGT1 family genes and BAHD acyltransferase genes using RiceXPro

To investigate the expression levels of OsGT1 family genes and BAHD acyltransferase genes in endosperm and embryos, the row expression-level data of RiceXPro (Sato *et al.*, 2011) were used. Normalization was performed as described previously (Ogo *et al.*, 2013). Hierarchical clustering was performed via Centroid Linkage Clustering in Cluster 3.0 using the gene-normalized value. Co-expression analysis with chalcone synthase gene (*OsCHS1*, Os11g0530600) was performed by RiceFREND (Sato *et al.*, 2012; http://ricefrend.dna.affrc.go.jp/). The glycosyltransferase genes in OsGT1, which were included in the top 100 of the co-expressed genes with *OsCHS1*, are shown in Supplementary Fig. S2C at *JXB* online).

### Extraction and measurement of free amino acids

Free amino acids were extracted with 5% (w/v) trichloroacetic acid at room temperature overnight from powder produced from mature rice kernels. The measurement of free amino acids by HPLC was performed as described by Kawakatsu *et al.* (2010).

### Fluorescent labelling of flavonoids in rice kernels

Fluorescent labelling was performed as described by Ogo *et al.* (2013), with or without the mild deglycosylation method (Hsieh and Huang, 2007), to investigate the subcellular localization of flavonoid glycosides as well as of flavonoid aglycones. Flavonoids and protein body-I (PB-I) were stained with diphenylboric acid 2-amino ethyl ester (DPBA) and rhodamine B, respectively. Protein body-II (PB-II) was immunostained with anti-OsTIP3 antibody (Os10g0492600; Kudo *et al.*, 2013) diluted 1:500, and Alexa Fluor 647-conjugated goat anti-mouse IgG (Invitrogen) diluted 1:500 was used as a specialized antibody. The labelled sections were examined with an epifluorescence microscope as described by Ogo *et al.* (2013).

## Results and discussion

### Analysis of metabolites of flavonoid rice seeds by LC-PDA-QTOF-MS

In transgenic flavonol, isoflavone, and flavone rice, *PAL*/*CHS*/*F3H*/*FLS*, *PAL*/*CHS*/*IFS*, and *PAL*/*CHS*/*FNS* are expressed under the control of the embryo- and endosperm-specific *18kDa oleosin* promoter in order to synthesize kaempferol, genistein, and apigenin, respectively (Ogo *et al.*, 2013). Metabolites were extracted from whole seeds (brown rice) of three independent lines of flavonol (lines 19, 6, and 17) and isoflavone (lines 12, 10, and 2) rice and two independent lines of flavone rice (lines 20 and 23). Furthermore the metabolites, were separately extracted from their endosperm and embryos to compare the flavonoid profiles between the endosperm and the embryos. The metabolites of non-transformant (NT) rice were extracted as a control. These extracts were subjected to LC-PDA-QTOF-MS.

**Fig. 1.**
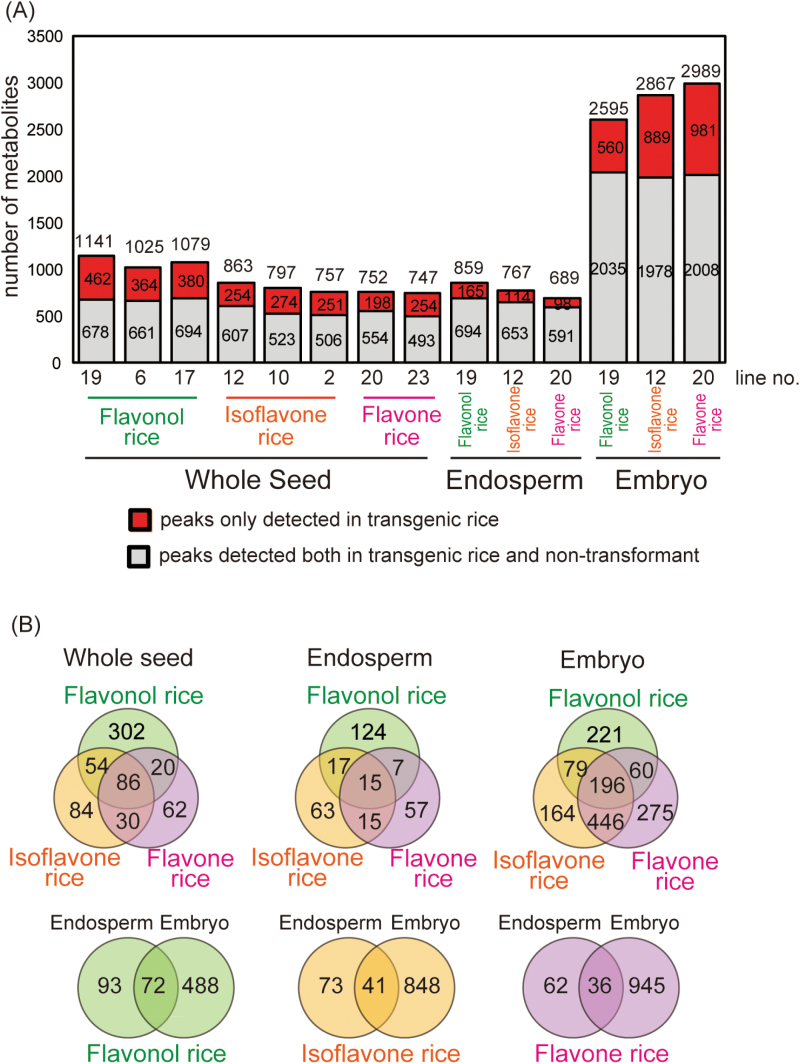
Analysis of all peaks detected by LC-PDA-QTOF-MS. (A) Number of peaks detected in transgenic rice and the non-transformant (NT). Numbers above the bars indicate the total number of peaks detected in each line. (B) Venn diagrams of the peaks only detected in transgenic rice. The peak data from line 19 of flavonol rice, line 12 of isoflavone rice, and line 20 of flavone rice were used.

A total of 4 392 peaks were detected in the extracts of transgenic flavonol, isoflavone, flavone, and non-transgenic (NT) rice. In whole seeds of transgenic flavonol, isoflavone, and flavone rice, 1 000–1 200, 700–900, and 700–800 peaks were detected, respectively (Fig. 1A). In the endosperm of transgenic rice, 600–900 peaks were detected, while 2 500–3 000 peaks were detected in the embryos of transgenic rice (Fig. 1A). Embryos are thought to contain higher concentrations of metabolites than whole seeds or endosperm and this was indeed detected under the present experimental conditions; the levels of many metabolites were below measurable limits in whole seeds and endosperm, while many more peaks were detected in embryos than in whole seeds or endosperm. Among the peaks detected in transgenic rice, 60–80% were also detected in NT rice, while the remaining 20–40% were only detected in transgenic rice (Fig. 1A). The peaks that were only detected in transgenic rice include flavonoids synthesized by exogenous enzymes introduced into transgenic rice and may also include intermediates, side-products, and degradation products of these flavonoids.

Among the peaks that were only detected in transgenic rice, 86, 15, and 196 peaks were found in whole seeds, endosperm, and embryos, respectively, of all three types of transgenic rice (flavonol, isoflavone, and flavone rice; Fig. 1B). A substantial number of peaks from all three types of transgenic rice overlapped, even though flavonols, isoflavones, and flavones were differently biosynthesized from flavanones by F3H/FLS, IFS, and FNS, respectively. Common peaks detected in all three types of transgenic rice included several flavones derived from apigenin, chrysoeriol, tricin, and luteolin (Fig. 4; see Supplementary Table S1 and Supplementary Fig. S1 at *JXB* online). Since rice bran contains small amounts of flavones, the endogenous enzymes involved in flavone biosynthesis are expected to be expressed in rice seeds. In flavonol and isoflavone rice, some flavanones synthesized by PAL and CHS may have been converted to flavones by endogenous flavone biosynthetic enzymes rather than converted by exogenous F3H/FLS and IFS, respectively.

Among the peaks that were detected only in transgenic rice, there were 72, 41, and 36 common peaks in transgenic flavonol, isoflavone, and flavone rice, respectively, in both endosperm and embryos (Fig. 1B). The newly synthesized metabolites in endosperm and embryos varied substantially even though the same exogenous enzymes were expressed, suggesting that the expression of endogenous enzymes involved in the degradation and modification of the metabolites synthesized in transgenic rice may be substantially different between endosperm and embryos.

**Fig. 2.**
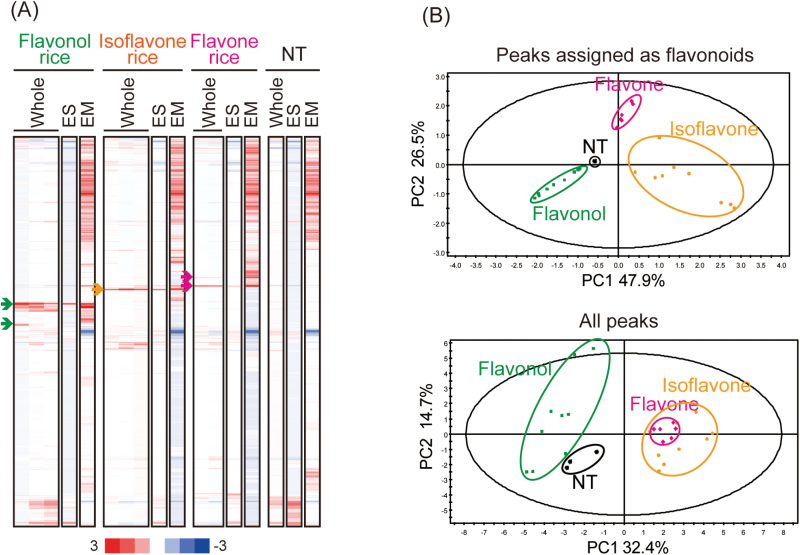
Cluster analysis and principal component analysis (PCA). (A) Hierarchical cluster analysis of all the metabolites detected in transgenic rice and the non-transformant (NT). Green, orange, and pink arrows indicate flavonol, isoflavone, and flavone rice-specific clusters, respectively. Whole, whole seeds; ES, endosperm; EM, embryos. (B) PCA using data from the peaks assigned as flavonoids and all the peaks detected in the present study. Flavonol rice, green squares; isoflavone rice; orange circles; flavone rice, pink squares; NT, black crosses.

An hierarchical cluster analysis of all peaks detected by LC-PDA-QTOF-MS (Fig. 2A) was performed, revealing flavonol, isoflavone, and flavone rice-specific clusters. These clusters include newly synthesized flavonoids by exogenous enzymes. A PCA of the processed data from whole seeds was performed (Fig. 2B). The scatterplots of the peaks (which were assigned as flavonoids, as described in the following paragraph) in flavonol rice, isoflavone rice, flavone rice, and NT are clearly separated. On the other hand, using all peaks, the scatterplots of isoflavone rice and flavone rice are well correlated, indicating that the two types of transgenic rice possess many common compounds.

### Characterization of flavonoids in transgenic rice by LC-PDA-QTOF-MS

**Fig. 3.**
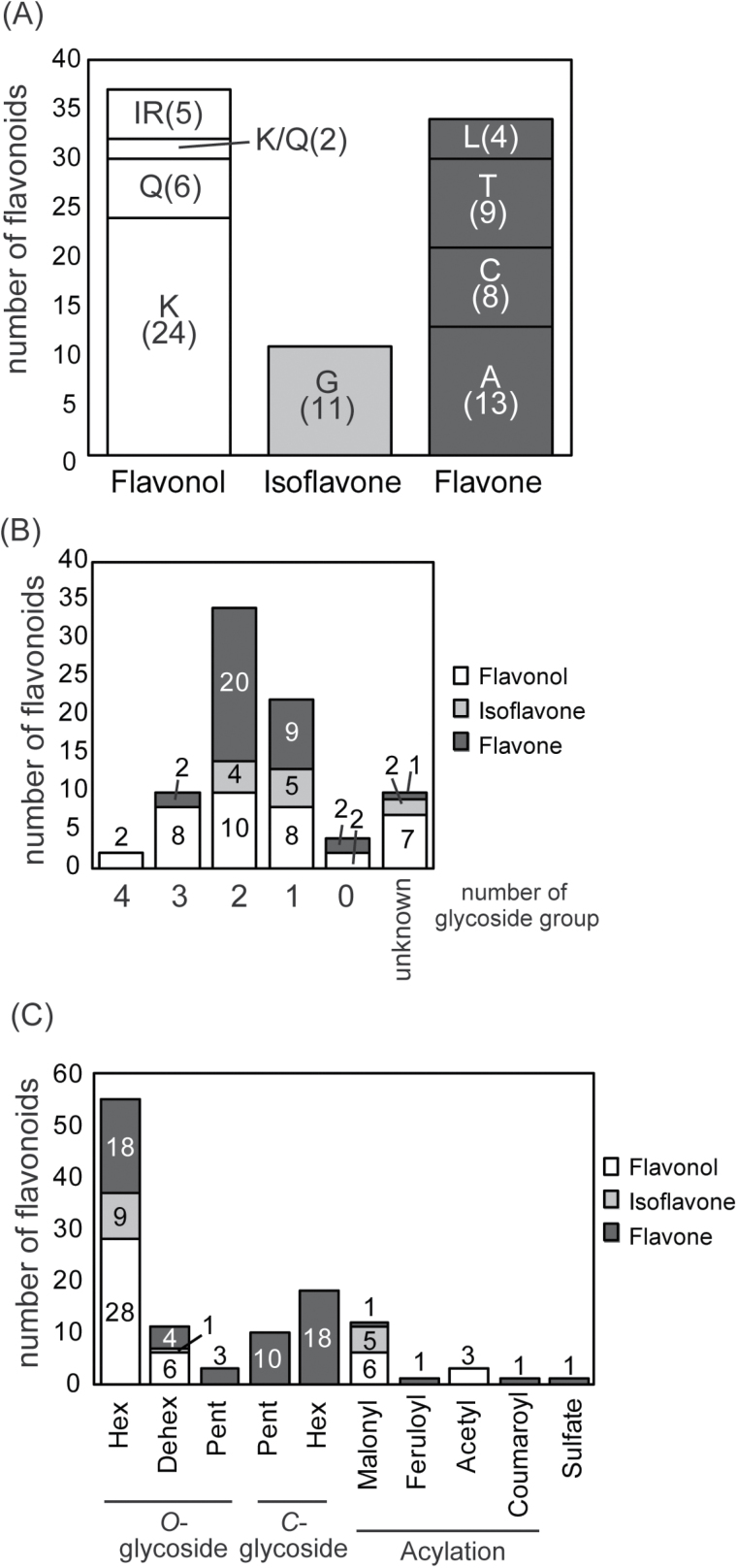
Investigation of aglycones, glycosylation, and acylation of flavonoids.(A) Number of flavonoids derived from kaempferol (K), isorhamnetin (IR), quercetin (Q), genistein (G), apigenin (A), chrysoeriol (C), tricin (T), and luteolin (L). (B) Number of flavonoids with one to four glycoside groups; without glycoside groups; or unknown composition. (C) Number of glycosylated, acylated, and sulphated flavonoids in transgenic rice. Hex, hexose; Dehex, deoxyhexose; Pent, pentose. (This figure is available in colour at *JXB* online.)

**Fig. 4.**
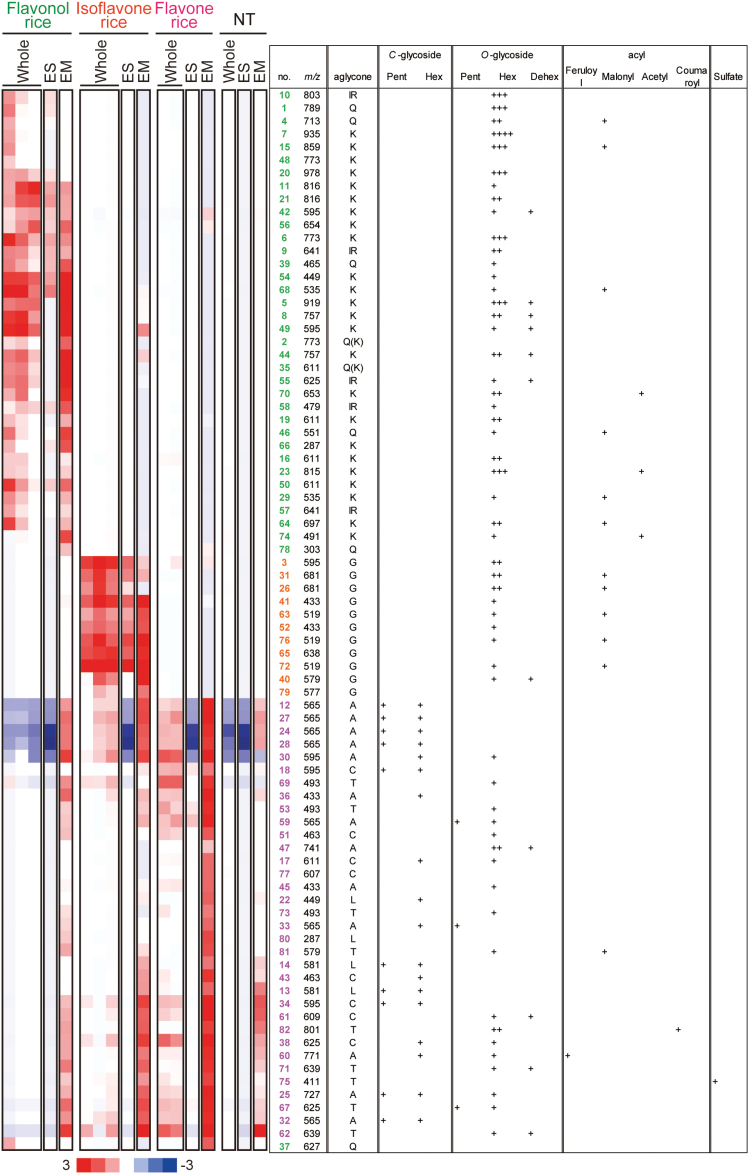
Hierarchical cluster analysis of peaks assigned as flavonoids in transgenic rice and the non-transformant (NT). Whole, whole seeds; ES, endosperm; EM, embryos; No, peak number; *m/z*, [M^+^H]^+^ molecular ion weight as given by MS in positive mode; Hex, hexose; Dehex, deoxyhexose; Pent, pentose; K, kaempferol; IR, isorhamnetin; Q, quercetin; G, genistein; A, apigenin; C, chrysoeriol; T, tricin; L, luteolin. In embryos of flavone rice, the intensity of compound **37** includes the intensity of the isotope of compound **38**.

Aglycones, glycosylation patterns, and acylation patterns based on accurate mass, retention time, PDA spectra, and MS/MS fragmentation data were then investigated. As a result, 82 compounds were chemically assigned as flavonoids, which included 37 flavonols, 11 isoflavones, and 34 flavones (Figs 3A, 4; see Supplementary Table S1 and Supplementary Fig. S1 at *JXB* online).

MS/MS spectra (in the positive mode) of 24, 6, and 5 compounds among the 37 compounds assigned as flavonol showed diagnostic fragment ions of kaempferol, quercetin, and isorhamnetin at *m/z* 287, 303, and 317, respectively (Fig. 3A; see Supplementary Table S1 at *JXB* online) (Yonekura-Sakakibara *et al.*, 2008). These MS/MS spectra, as well as the PDA spectra of these compounds, indicate that they were kaempferol, quercetin, and isorhamnetin derivatives or aglycones, respectively. Compounds **66** and **78** were assigned as kaempferol and quercetin, respectively, since their MS/MS spectra, which did not show fragment ions corresponding to sugar or acyl moieties, matched well with the fragmentation patterns of kaempferol and quercetin in MassBank (Horai *et al.*, 2010). The aglycones of compounds **2** and **35** could be assigned as kaempferol and quercetin derivatives based on their MS/MS and PDA spectra. Although the most common aglycone form of flavonols in flavonol rice was kaempferol, some quercetin and isorhamnetin derivatives were also detected. These quercetin and isorhamnetin derivatives may have been synthesized from kaempferol by endogenous hydroxylases and methyltransferases.

All 11 compounds assigned as isoflavones showed diagnostic PDA spectra and fragment ions at *m/z* 271 of genistein, indicating that these compounds were genistein derivatives (Fig. 3A; see Supplementary Table S1 at *JXB* online) (Eunmi and Alyson, 2011). Unlike flavonol rice, only isoflavone glycosides with genistein as an aglycone were detected in isoflavone rice, suggesting that endogenous hydroxylases and methyltransferases in rice do not catalyse genistein as substrates.

Among the 34 compounds assigned as flavones, three, three, nine, and one compound showed diagnostic fragment ions of apigenin, chrysoeriol, tricin, and luteolin at *m/z* 271, 301, 331, and 287, respectively (Iwashina *et al.*, 2010). These MS/MS spectra and the PDA spectra indicate that these compounds were apigenin, chrysoeriol, tricin, and luteolin derivatives or aglycones, respectively. Compound **80** was assigned as luteolin because the MS/MS spectrum matched well with the fragmentation pattern of luteolin in MassBank (Horai *et al.*, 2010). On the other hand, the other 18 compounds assigned as flavones did not show diagnostic fragment ions of apigenin, chrysoeriol, tricin, or luteolin in their MS/MS spectra. The MS/MS spectra of these compounds showed diagnostic fragmentation patterns of *C*-glycosides (see Supplementary Table S1 at *JXB* online), which do not include fragment ions of aglycones. These MS/MS spectra and the PDA spectra indicate that these compounds were apigenin, chrysoeriol, tricin, and luteolin derivatives with *C*-glycosylation. In total, 13 apigenin, eight chrysoeriol, nine tricin, and four luteolin derivatives were detected in flavone rice (Fig. 3A). Rice bran and embryos contain a few flavones derived from apigenin, chrysoeriol, and tricin (Matsuda *et al.*, 2012; Galland *et al.*, 2014), suggesting that endogenous hydroxylases and methyltransferases, which efficiently catalyse flavones, are expressed in seeds, especially in embryos and the aleurone layer. A substantial portion of apigenin synthesized by exogenous enzymes is thought to be hydroxylated and/or methoxylated by endogenous hydroxylases and methyltransferases.

### Characterization of flavonoid glycosylation and acylation

In MS/MS experiments analysing *O*-glycosylated flavonoids, fragmentation is easily induced by the loss of hexose (*m/z* 162), deoxyhexose (*m/z* 146), and pentose (*m/z* 132) from the precursor ion (Cuyckens and Claeys, 2004). On the other hand, the *C*-glycosidic bond of flavonoids is not easily cleaved by weak voltage to ionize compounds under MS. The loss of water molecules and cross-ring cleavages of sugar residues are characteristic fragments of *C*-glycosides (Cuyckens and Claeys, 2004). In the present analysis, many flavonoids showed the characteristic fragmentation patterns of *O*-glycoside and *C*-glycoside. Based on these fragmentation patterns in the MS/MS spectra, 69 *O*- and/or *C*-glycosylated flavonoids were characterized among the 82 flavonoids. Flavonols, isoflavones, and flavones were glycosylated with one to four, one to two, and one to three sugar moieties, respectively, in individual transgenic rice (Figs 3B, 4; see Supplementary Table S1 at *JXB* online). Most flavonols and isoflavones were *O*-glycosylated with hexoses, while some were *O*-glycosylated with deoxyhexoses (Figs 3C, 4; see Supplementary Table S1 at *JXB* online). On the other hand, many flavones were not only *O*-glycosylated with hexoses, deoxyhexoses, and/or pentoses but were also *C*-glycosylated with hexoses and/or pentoses. The MS/MS fragmentation patterns also reveal acylation and sulphation of the flavonoids. In the MS/MS analysis examining malonyl, feruloyl, acetyl, coumaroyl, and sulphated flavonoids, the *m/z* of the fragmentation ion reflected the conjugation of malonyl (*m/z* 86 or malonylhexose; *m/z* 248), feruloyl (*m/z* 176), acetyl (acetylhexose; *m/z* 204), coumaroyl (coumaroylhexose; *m/z* 308), and sulphate (*m/z* 80) moiety, respectively (Cuyckens and Claeys, 2004; Galland *et al*., 2014). Twelve malonyl flavonoids, one feruoyl flavone, three acetyl flavonols, one coumaroyl flavone, and one sulphated flavone were detected in the transgenic rice seeds (Figs 3C, 4). These glycosylated, acylated, and sulphated flavonoids are thought to be biosynthesized by endogenous enzymes in rice from new flavonoids synthesized by the exogenous enzymes.

Among the flavones, the fragmentation patterns of compounds **60**, **62**, and **71** matched well with those of compound **27** [isovitexin 2′′-*O*-(6′′′-(*E*)-feruloyl)-glucopyranoside], compound **4** (tricin 7-*O*-rutinoside), and compound **5** (tricin 7-*O*-neohesperidoside) reported by Yang *et al.* (2014), respectively (see Supplementary Table S1 at *JXB* online). Hence, compounds **60**, **62**, and **71** were annotated as isovitexin 2′′-*O*-(6′′′-(*E*)-feruloyl)-glucopyranoside, tricin 7-*O*-rutinoside, and tricin 7-*O*-neohesperidoside, respectively.

Glycosylation of flavonoids modulates their chemical and physical parameters, such as solubility, stability, and membrane permeability. *O*-linked glycosylation can enhance certain types of biological benefits such as anti-rotavirus and anti-obesity activity, while *C*-linked gycosylation improves biological activities such as anti-diabetic activity (Bae *et al.*, 2000; Matsuda *et al.*, 2003; Choi *et al.*, 2014). The introduction of acyl groups also enhances physical parameters such as thermo-stability and light-resistivity as well as biological effects such as antioxidant and antimicrobial activity (Fossen *et al.*, 1998; Ishihara and Nakajima, 2003; Mellou *et al.*, 2005). Such flavonoid modifications affecting bioactivity increase the diversity of pharmacokinetic behaviours and biological benefits. Furthermore, because flavonoid biosynthetic enzymes are heterologously and ectopically expressed in rice seeds in our transgenic lines, novel, non-natural flavonoid glycosides may be biosynthesized by new combinations of exogenous flavonoid biosynthetic enzymes and endogenous enzymes that function in glycosylation/acylation in the transgenic rice. In the present study, it has been demonstrated that flavonoids with the intended aglycones were efficiently produced in rice seeds by introducing exogenous gene constructs for flavonoid biosynthesis, while these flavonoids were variably *O*-glycosylated, *C*-glycosylated, acylated, and sulphated. Use of this method, i.e. heterologous and ectopic expression of biosynthetic enzymes, expands the diversity of flavonoid structures and leads to the production of novel, hybrid, physiologically active substances, providing useful resources as seed compounds for drug discovery.

### Accumulation patterns of flavonoids

A hierarchical cluster analysis of the 82 flavonoids in transgenic rice was performed based on intensity. Three clusters were produced, i.e. the flavonol, isoflavone, and flavone rice-specific clusters, which included flavonols, isoflavones, and flavones, respectively (Fig. 4). The whole seeds of three independent lines of flavonol and isoflavone rice and two independent lines of flavone rice accumulated almost the same levels of flavonoids. Higher levels of several flavonols, and most flavones, were present in embryos compared with the endosperm, while similar concentrations of several flavonols, and most isoflavones, were detected in the endosperm and embryos (Fig. 4). Even though the genes encoding all of the exogenous enzymes were expressed under the control of the *oleosin* promoter in an embryo- and endosperm-specific manner in transgenic flavonol, isoflavone, and flavone rice, the accumulation patterns of the flavonoids differed in the endosperm and embryos depending on the class of flavonoid and the glycosylation/acylation pattern. These results suggest that the efficiency of flavonoid synthesis, modification (such as glycosylation and acylation), transport, and accumulation differs among flavonoid classes and glycosylation/acylation patterns, reflecting the difference in net flavonoid concentrations in the endosperm and embryos.

Glycosyltransferases in the OsGT1 family are involved in the glycosylation of small molecules including flavonoids (Cao *et al.*, 2008). BAHD acyltransferases catalyse the acylation of various kinds of molecules including flavonoids (Tuominen *et al.*, 2011). The expression patterns of OsGT1 family and BAHD acyltransferase genes were examined using RiceXpro (Sato *et al.*, 2011; http://ricexpro.dna.affrc.go.jp/). As a result, glycosyltransferase and acyltransferase genes expressed only in endosperm, only in embryos, and in both endosperm and embryos, respectively were found (see Supplementary Fig. S2A, B at *JXB* online). Furthermore, to narrow down the enzymes involved in the glycosylation and acylation of flavonoids, co-expression profiling with chalcone synthase (*OsCHS1*, Os11g0530600), which is a key enzyme involved in biosynthesis of flavonoids, was used. The public database, RiceFREND (Sato *et al.*, 2012;http://ricefrend.dna.affrc.go.jp/) was used which is a gene co-expression database in rice based on a large collection of microarray data derived from various tissues/organs at different stages of growth and development under natural field conditions, and rice plants treated with various phytohormones. Analysis of RiceFREND showed that several glycosyltransferase genes in the OsGT1 family were co-expressed with *OsCHS1*. All of them were expressed in the embryo, while two genes (Os04g0305700, Os02g0589400) were also expressed in the endosperm (see Supplementary Fig. S2C at *JXB* online). These glycosyltransferases may be involved in the glycosylation of flavonoids in endosperm and/or embryo of transgenic rice seeds. Among OsGT1s’ *C*-glycosyltransferases, OsCGT catalyses the *C*-glucosylation of flavonoids in rice (Brazier-Hicks *et al.*, 2009). Three homologues of OsCGT were identified in the Rice GT database (Cao *et al.*, 2008). *OsCGT* and one homologue of the *OsCGT* are expressed in endosperm and the other two homologues are expressed in embryos (see Supplementary Fig. S2D at *JXB* online). Although OsCGT and its homologues were not strongly correlated with *OsCHS1*, these endogenous *C*-glycosyltransferases are thought to catalyse *C*-glycosylation of flavones synthesized by the exogenous enzymes in a tissue-specific manner. Although BAHD acyltransferase genes, which were co-expressed strongly with chalcone synthase, could not be found by RiceFREND, a flavonoid malonyltransferase, OsMaT-2, was expressed in the endosperm and embryo of rice seed (see Supplementary Fig. S2B at *JXB* online). Because OsMaT-2 utilizes flavonol glucosides, isoflavone glucosides, and flavone glucosides as substrates (Kim *et al.*, 2009), it may be involved in the malonylation of flavonoids in the seeds of flavonol, isoflavone, and flavone rice plants. The expression patterns of these glycosylation and acylation enzymes may lead to the different accumulation of flavonoids with various glycosylation and/or acylation patterns in endosperm and embryos.

### Free amino acid contents

To examine the free amino acid profile, amino acids were extracted from whole seeds, and subjected to HPLC. The amounts of phenylalanine and tyrosine, which are starting molecules of flavonoid biosynthesis, are almost the same among the three transgenic and the NT rice (see Supplementary Fig. S3 at *JXB* online). 100–200 nmol g^–1^ DW of phenylalanine and tyrosine accumulated in the seeds, while the transgenic rice contained 2 000 nmol g^–1^ DW of flavonols, 1 000 nmol g^–1^ DW of isoflavones, and 150 nmol g^–1^ DW of flavones, respectively (Ogo *et al.*, 2013). The flavonoids synthesized by the exogenous enzymes accumulated much more or at the same level compared with the phenylalanine and tyrosine. This result suggests that the phenylalanine and tyrosine are synthesized immediately after consumption for flavonoid biosynthesis, and that constant amounts of the free phenylalanine and tyrosine are maintained in rice seed.

Unexpectedly, the amounts of free asparagine, arginine, and lysine were increased in flavonol rice (see Supplementary Fig. S3 at *JXB* online). These amino acids are not directly related to flavonoid synthesis. Le Gall *et al.* (2003) reported that the levels of some metabolites such as arginine, which are not directly associated with flavonoids biosynthesis, were different between NT tomato and transgenic tomato which contains increased levels of flavonol on introducing the maize transcription factors *LC* and *C1*. It is well-known that metabolite contents are greatly affected by environmental parameters such as the soil nutrients, the climate, the season, etc. Although the transgenic and NT rice were grown side-by-side in a greenhouse under the same conditions, the accumulation of flavonoids synthesized by the exogenous enzymes may elicit changes of cellular environment and affect the biosynthesis and accumulation of the metabolites which are not directly associated with flavonoid biosynthesis, such as asparagine, arginine, and lysine. In addition, many kinds of flavonoid could perform as signal molecules to affect plant development and result in enormous changes of gene expression profiles such as the jasmonate biosynthetic genes (Pourcel *et al.*, 2013). In transgenic rice seeds, flavonoids biosynthesized by exogenous enzymes may act as signal molecules to affect the amino acids’ biosynthetic pathway.

### Subcellular localization of flavonoids in endosperm

**Fig. 5.**
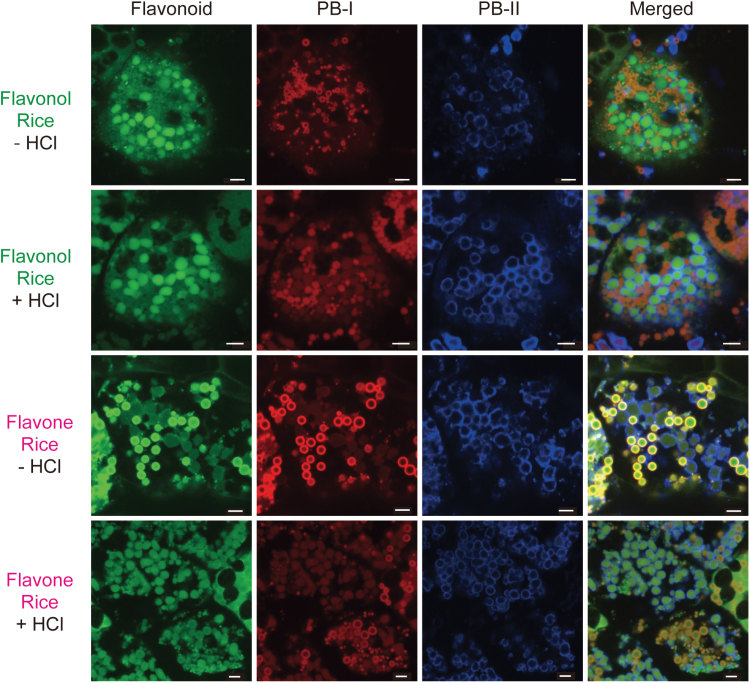
Subcellular localization of flavonoids in the endosperm of flavonol rice and flavone rice. Maturing seeds of transgenic rice were triple-stained with diphenyl boric acid 2-amino ethyl ester (DPBA), which stains flavonoids (‘Flavonoid’ column, green), rhodamine B which stains protein body-I (‘PB-I’ column, red), and anti-OsTIP3 antibody which stains protein storage vacuoles (‘PB-II’ column, blue). Merged images are shown in the right column (‘Merged’ column). –HCl and +HCl indicate without and with deglycosylation, respectively. Bars=5 μm. Line 19 of flavonol rice and line 20 of flavone rice were used.

Rice endosperm contains two types of protein body that differ in size, shape, electron density, and origin, called ER-derived PB-I which contains prolamins, and protein storage vacuole (PB-II) which contains glutelins and 26kDa globulin, respectively (Takaiwa *et al.*, 1999). In a previous study, it was demonstrated that flavonols in flavonol rice are deposited in PB-II in the endosperm, as revealed using DPBA, a flavonoid-specific dye (Ogo *et al.*, 2013). DPBA reacts with non-glycosylated flavonoids and emits fluorescence under UV irradiation. The fluorescence of DPBA-glycosylated flavonoids is weaker than that of DPBA-non-glycosylated flavonoids, while flavonol rice seeds react with DPBA without a prior deglycosylation treatment, suggesting that most fluorescence is derived from flavonol aglycones. In the current study, to investigate the subcellular localization of flavonoid glycosides, a mild deglycosylation method was used to observe flavonoids in rice seeds (Hsieh and Huang, 2007). In flavonol rice, DPBA fluorescence was observed in PB-II with and without deglycosylation, suggesting that both flavonol aglycones and glycosides are deposited in PB-II (Fig. 5). In flavone rice, DPBA fluorescence was observed in PB-I without deglycosylation (Fig. 5), indicating that flavone aglycones were deposited in PB-I. On the other hand, DPBA fluorescence was observed in both PB-I and PB-II with deglycosylation. Flavone glycosides are thought to be deposited in PB-II, or in both PB-I and PB-II, in flavone rice endosperm. In flavonol rice and flavone rice endosperm, no strong signals of flavonoids were observed in other organelles (such as amyloplasts and the nucleus) than PB-I and PB-II. In isoflavone rice, DPBA fluorescence was too weak for the detection of isoflavone signals.

Consistent with their diverse physiological functions, flavonoids are found in most plant cellular compartments, including vacuoles, ER-derived vesicles, and nuclei, as well as extracellular spaces (Onyilagha and Grotewold, 2004; Saslowsky *et al.*, 2005; Hsieh and Huang, 2007; Naoumkina *et al.*, 2007). Most conjugated flavonoids, such as anthocyanins, flavonol, and flavone glycosides, are found primarily in the vacuole (Winkel-Shirley, 2001; Frangne *et al.*, 2002; Dixon, *et al.*, 2005; Lepiniec *et al.*, 2006). Glycosylation of some flavonoids is essential for their transport into the vacuole by multidrug and toxin extrusion (MATE) transporters (Matern *et al.*, 1983, 1986; Marinova *et al.*, 2007). In addition, MRP-type ABC transporters are involved in the transport of anthocyanins and glycosylated flavones to the vacuole (Klein *et al.*, 2000; Goodman *et al.*, 2004; Francisco *et al.*, 2013). Acylation is also involved in flavonoid transport to and retention within the vacuole (Matern *et al.*, 1983; Hopp and Seitz, 1987). In the present study, it was found that glycosylated/acylated flavonols were deposited in PB-II, and glycosylated/acylated flavones were deposited in PB-II or PB-I/PB-II (Fig. 5). Glycosylation and acylation of these flavonols and flavones may facilitate their transport to PB-II, which is derived from the vacuole, via endogenous MATE and ABC transporters. Interestingly, flavonol aglycones were also deposited in PB-II (Fig. 5). Apart from these transporters, which require glycosylation and acylation for their transport activity, flavonoids are thought to be transported to the vacuole via prevacuolar compartments (Zhang *et al.*, 2006). These flavonol aglycones may be transported to PB-II via different pathways from those used by MATE and ABC transporters, such as through prevacuolar compartments. In contrast to flavonol aglycones, flavone aglycones were deposited in PB-I (Fig. 5). While in a few studies, flavonoid aglycones (such as quercetin and kaempferol) were observed in the nuclear region, plasma membrane, and endomembrane system (Peer *et al.*, 2001), little additional information is available about the subcellular localization of flavonoid aglycones. The present study demonstrates that flavonol and flavone aglycones are transported to (and deposited in) vacuole- and ER-derived vesicles, respectively, providing useful information about the mechanisms underlying the transport and deposition of flavonoid aglycones.

## Conclusion

Metabolome analysis using LC-PDA-QTOF-MS, chemically assigned 82 flavonoids in transgenic rice seeds which were variously glycosylated and acylated. Flavonoids synthesized by exogenous enzymes in rice seeds were modified by endogenous enzymes and subsequently trafficked, resulting in stable accumulation in PB-I and/or PB-II. These results suggest that heterologous and ectopic expression of biosynthetic enzymes in rice seeds is not only an efficient production platform for flavonoids, but it can also be used to expand the structural diversity of flavonoids and, therefore, represents a novel, unexplored source of bioactive molecules. The present method may be useful for producing other economically valuable metabolites that are scarce and currently expensive to produce.

## Supplementary data

Supplementary data can be found at *JXB* online.

Supplementary Table S1. List of flavonoids assigned by LC-PDA-QTOF-MS in transgenic rice.

Supplementary Fig. S1. Representative chromatogram of transgenic and non-transformant (NT) rice at 340nm.

Supplementary Fig. S2. Expression profiles of glycosyltransferase genes in the OsGT1 family and the BAHD acyltransferases which are involved in glycosylation and acylation, respectively, of various kinds of molecules including flavonoids.

Supplementary Fig. S3. Free amino acid contents in non-transformant (NT) and transgenic rice seeds.

Supplementary Data

## Abbreviations:

CGT: *C*-glycosyltransferases
CHS: chalcone synthase
DPBA: diphenylboric acid 2-amino ethyl ester
F3H: flavanone 3-hydroxylase
FLS: flavonol synthase
IFS: isoflavone synthase
FNS: flavone synthase
LC-MS/MS: liquid chromatography tandem mass spectrometry
LC-PDA-QTOF-MS: liquid chromatography-photodiode array-quadrupole time-of-flight mass spectrometry
MATE: multidrug and toxin extrusion
NT: non-transformant
PAL: phenylalanine ammonia lyase
PB-I: protein body-I
PB-II: protein body-II
PCA: principal component analysis.
