# Supplementary material for: Transgenic rice seed expressing flavonoid biosynthetic genes accumulate glycosylated and/or acylated flavonoids in protein bodies
Source: J Exp Bot. 2015 Oct 4;67(1):95–106. doi: 10.1093/jxb/erv429 (PMC4682426; doi:10.1093/jxb/erv429)
Supplement: Supplementary Data [file supp_67_1_95__index.html]

Transgenic rice seed expressing flavonoid biosynthetic genes accumulate glycosylated and/or acylated flavonoids in protein bodies — Supplementary Data 

# Transgenic rice seed expressing flavonoid biosynthetic genes accumulate glycosylated and/or acylated flavonoids in protein bodies

## Supplementary Data

Data files

- Supplementary Data - Supplementary Data
- Supplementary Data - Supplementary Data
