# Supplementary material for: Transgenic rice seed expressing flavonoid biosynthetic genes accumulate glycosylated and/or acylated flavonoids in protein bodies
Source: J Exp Bot. 2015 Oct 4;67(1):95–106. doi: 10.1093/jxb/erv429 (PMC4682426; doi:10.1093/jxb/erv429)

Supplementary Table Legends

Supplementary Table S1. List of flavonoids assigned by LC-PDA-QTOF-MS in transgenic rice

Green, orange, and pink compound numbers indicate flavonol, isoflavone, and flavone, respectively. *m.z.*, [M+H]+ molecular ion weight, as given by MS in positive mode; Pent, pentose; Hex, hexose; Dehex, deoxyhexose; K, kaempferol; IR, isorhamnetin; Q, quercetin; G, genistein; A, apigenin; C, chrysoeriol; T, tricin; L, luteolin.

[a]Calculation conditions (each element, C, H, and O; mass accuracy <10 mDa; charge, 1).

[b]Aglycone was determined using biological sample data and/or PDA.

Supplementary Figure Legends

Supplementary Figure S1

Representative chromatogram of transgenic and non-transformant (NT) rice at 340 nm. See Figure 4 and Supplementary Tables S1 for details of the detected peaks. Green, orange, and pink numbers indicate flavonols, isoflavones, and flavones, respectively. Dashed lines indicate common peaks in more than two transgenic rice lines or NT.

Supplementary Figure S2

Expression profiles of glycosyltransferase genes in the OsGT1 family and the BAHD acyltransferases, which are involved in glycosylation and acylation, respectively, of various kinds of molecules including flavonoids.

(A) All members of the OsGT1 family. (B) All members of the BAHD acyltransferases. *OsMaT-2* is a flavonoid malonyltransferase, whose substrates includes flavonol,

isoflavone, and flavone. (C) The genes in the OsGT1 family, which were included in top 100 of co-expressed genes with *OsCHS1*, are shown. (D) *C*-glycosyltransferases including *OsCGT* and homologous genes (*OsCGT-like*). DAF indicates “days after flowering”.

Supplementary Figure S3

Free amino acid contents in non-transformant (NT) and transgenic rice seeds. Error bars indicate the SD of three replicates. Green, orange, pink, and gray bars indicate flavonol rice, isoflavone rice, flavone rice, and NT, respectively.

Supplementary Figure S1

Whole seed

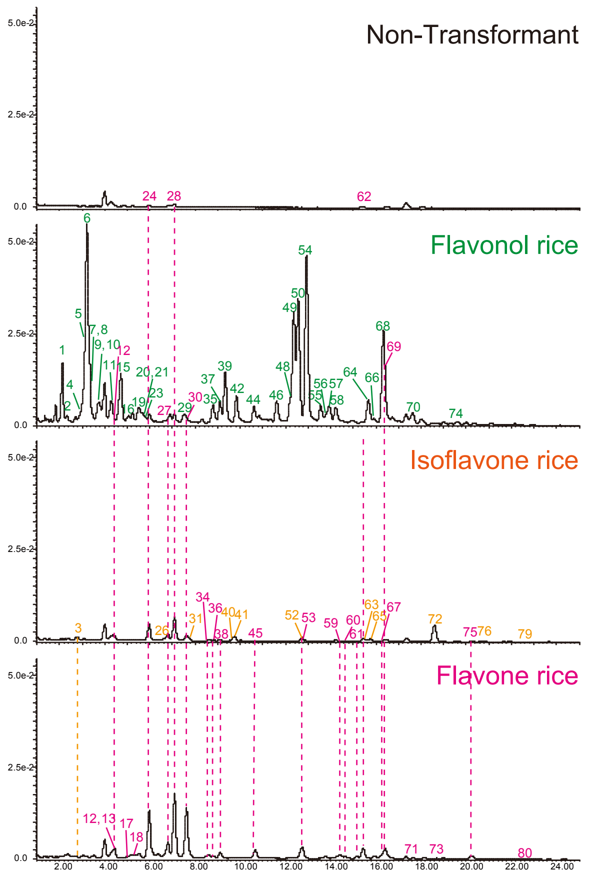

Endosperm

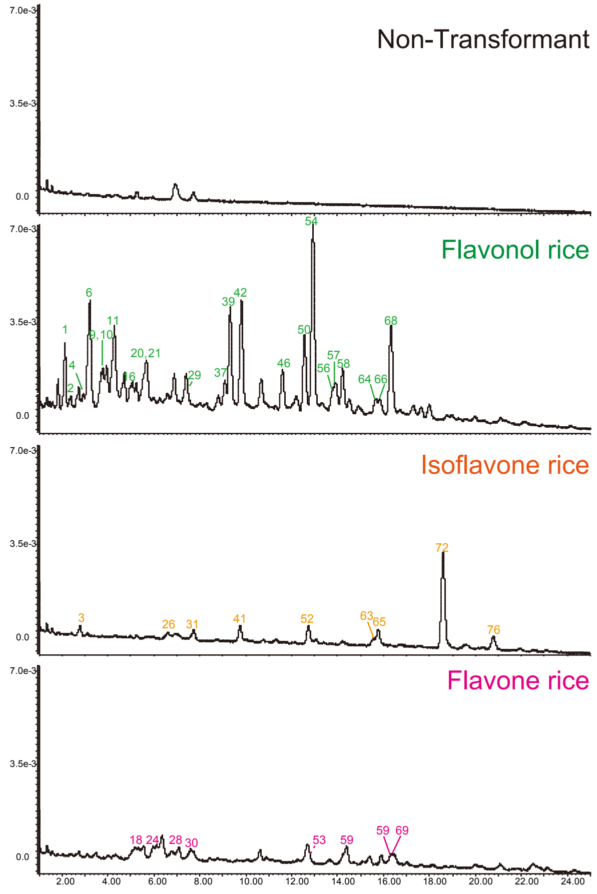

Embryo

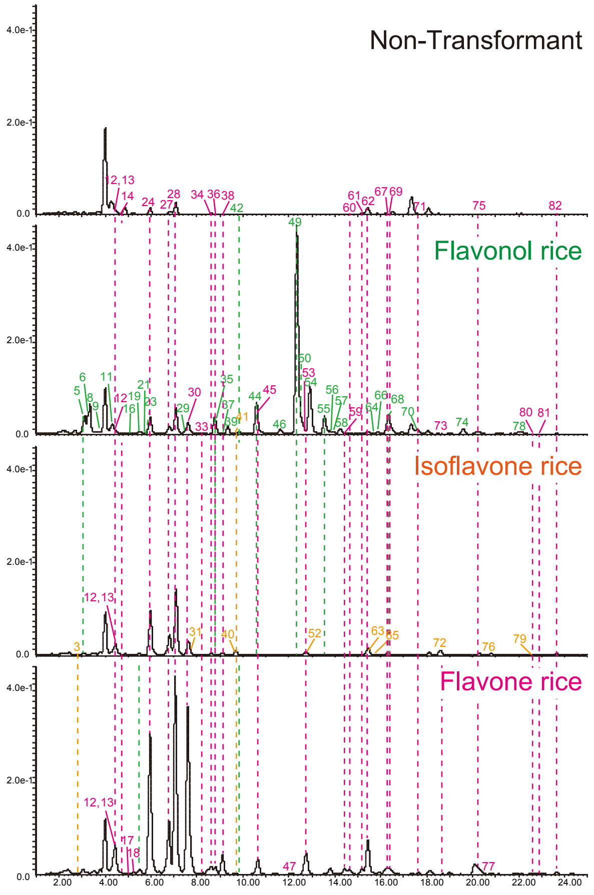

Supplementary Figure S2

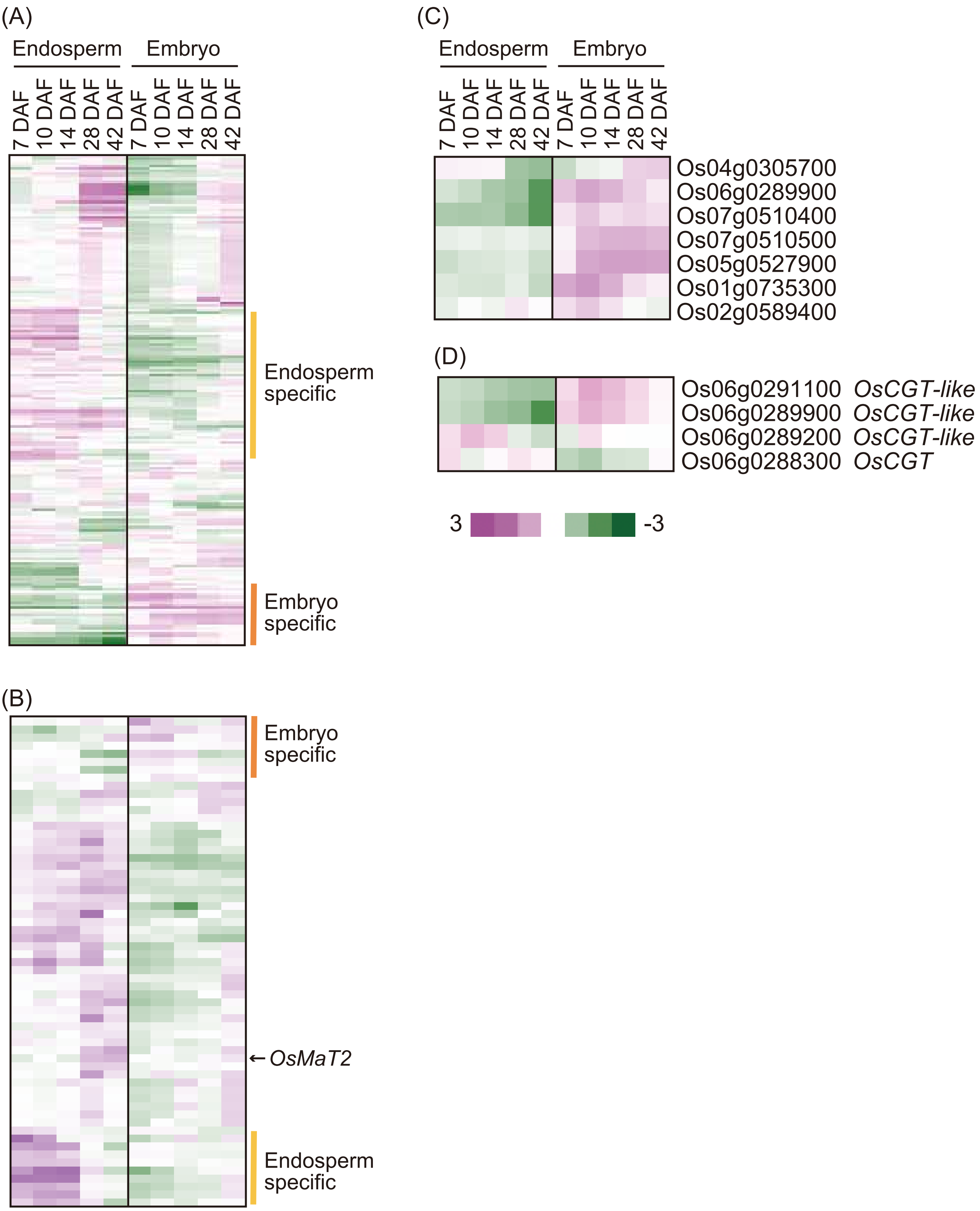

Supplementary Figure S3

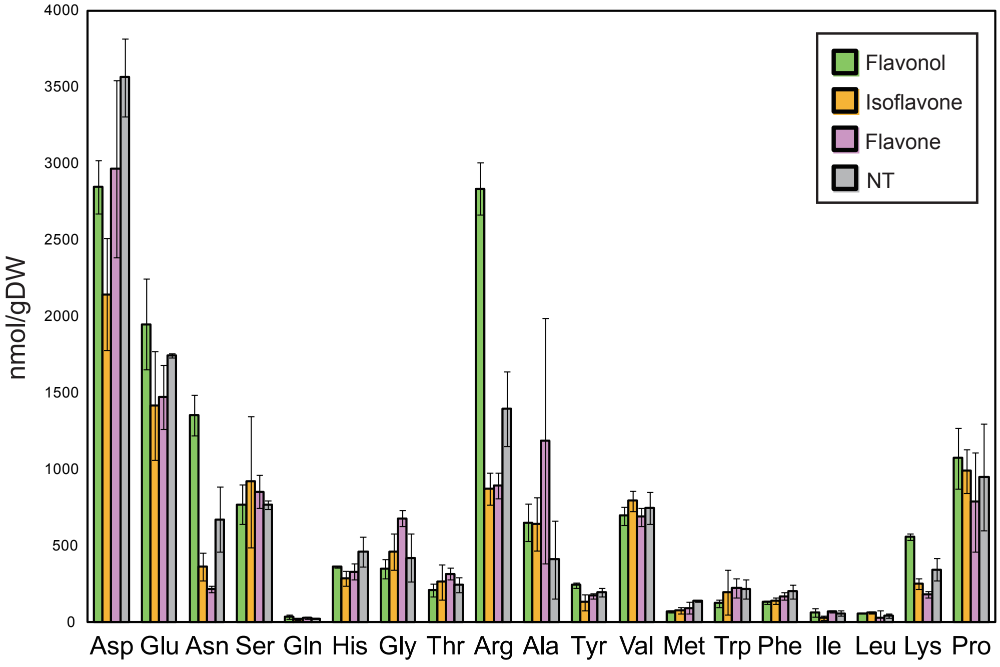

Supplement: Supplementary Data [file supp_erv429_Supplementary_data.pdf]
